# Perianal Fistula; from Etiology to Treatment - A Review

**DOI:** 10.34172/mejdd.2024.373

**Published:** 2024-04-30

**Authors:** Masoudreza Sohrabi, Somayeh Bahrami, Mozhdeh Mosalli, Mohsen Khaleghian, Mobin Obaidinia

**Affiliations:** ^1^Gastrointestinal and liver Diseases Research Center, Iran University of Medical Sciences, Tehran, Iran; ^2^Department of General Surgery, School of Medicine, Iran University of Medical Sciences, Tehran, Iran

**Keywords:** Anal fistula, Treatment, Surgery, Fistula tract

## Abstract

Anal fistula has been a challenging clinical issue for years due to its complex pathogenesis. The risk of frequent recurrence and incontinence complicates long-term treatment. Recent scientific literature has reviewed new techniques used for anal fistula treatment in recent years, assessing the advantages and disadvantages of each based on clinical outcomes. Although surgery is the main method used to treat anal fistula, there is no simple technique that can completely heal complex anal fistula. The surgical treatment should consider the healing outcome and the protection of anal function comprehensively. Several innovative techniques have emerged in recent years, such as combined techniques based on drainage seton and LIFT-plug, which appear to be relatively effective therapies. However, more multi-center prospective trials with long-term follow-up are needed to validate their effectiveness. In some situations, medical treatment may also be considered.

## Introduction

 A fistula-in-ano, also known as a perianal or anoperineal fistula, refers to an abnormal tract lined with epithelial tissue that connects the anal canal to the skin surrounding the anus.^1^ In this condition, the abnormal opening is located outside of the sphincter muscle complex, resulting in limited voluntary sphincter muscle fibers surrounding it.^2^ Perianal fistulas can be classified into two main categories,^3,4^ primary, which is caused by the obstruction of anal glands leading to stasis and infection with abscess, and secondary, which can be attributed to conditions such as inflammatory bowel diseases (IBDs), malignancy, iatrogenic factors, and infection.

 The primary cause of fistulas is often an anorectal abscess. In fact, the most common cause of fistulas is an anorectal abscess.^5,6^ Between 30% to 70% of patients who have an anorectal abscess also have a fistula-in-ano at the same time. For those who do not initially have a fistula, approximately one-third will eventually be diagnosed with a fistula-in-ano in the months to years following drainage of the abscess.^5,6^ The classification of fistulas is determined based on their location in relation to the anal sphincters.^7,8^

 Perianal fistulas are commonly observed in patients with IBD, particularly those with Crohn’s disease (CD). In this category, the diagnosis may be delayed if perianal fistulas develop before intestinal symptoms. However, if patients experience recurrent perianal abscesses, multiple complex fistulas, or have minimal symptoms despite the severity and diversity of perianal lesions, perianal fistulas should be suspected and promptly diagnosed.

 Although fistula-in-ano is typically a non-cancerous condition, it can cause considerable discomfort and even psychological problems for the patient. The condition can severely impact a patient’s quality of life and may also have a negative effect on their psychological state, leading to symptoms of depression or anxiety.^6,9^ The primary goal of treatment is to manage the infection and ensure fecal continence. Numerous treatment options are available, with new approaches continually being developed and evaluated.^3,4,10^ This article will provide an overview of the basic principles of diagnosing and treating fistula-in-ano (Box 1).

**Box 1. Approach to patients with perianal fistula**

**Diagnostic approach:**

Physical examination/Examination under anesthesia

Endoscopy: Evaluation Intestinal Inflammation, stenosis, internal fistula opening,

Imaging studies: EUS, MRI, TPUS

**Treatment approach:**

**Surgical:**

Abscess drainage

Seton

Fistulotomy

Fibrin glue

Lift

Fistula plug

Endorectal FLAP

Stem cell

Defunctioning of colon

**Medical:**

Antibiotics

Calcineurin inhibitors

Thiopurines

Anti-TNF-alpha

**Combination therapies**

## Epidemiology

 Fistula-in-ano is a common anorectal condition, more prevalent in men (12.3 cases per 100 000) than women (5.6 cases per 100 000), typically diagnosed around the age of 38, with a peak occurrence between 20 and 40 years of age.^11,12^ IBD, notably CD, is a primary cause of anal fistulas, with CD showing a significantly higher incidence of perianal fistulas (34%) compared with ulcerative colitis (UC) (4%).^12,13^ In some cases, perianal fistulas can present as the first symptom in patients with CD, sometimes preceding the onset of intestinal symptoms by several years.^13,14^ In patients with CD, perianal fistulas can manifest as the initial symptom even years before intestinal symptoms. About 25% of global CD cases exhibit perianal lesions, with 18% involving penetrating conditions like fistulas or abscesses. The prevalence of perianal CD rises with disease duration.^15^ A French study by Brochard and colleagues revealed cumulative probabilities of perianal CD occurrence at 1, 5, and 10 years to be 22%, 29%, and 32%, respectively. Additionally, cumulative probabilities of the fistulizing CD at 1, 5, and 10 years were 11%, 16%, and 19%, respectively.^16^ In a Danish Cohort study of 9739 patients with CD, perianal disease developed in 19%. These patients, compared with non-perianal CD patients were at increased risk of undergoing major surgery.^17^ In this context, a cohort study from South Korea shows a higher incidence of perianal CD and its complications compared with Western countries.^18^ Recent meta-analysis on the epidemiology of perianal CD indicated that approximately 1 in 5 patients with CD developed perianal disease within 10 years of disease.^15^ A study at the Mayo Clinic on 414 patients noted a cumulative incidence of perianal disease of 24% over 30 to 40 years.^19^ Göttgens et al conducted a study in the Netherlands to evaluate anal fistulas in 1162 patients with CD. They found that the incidence of anal fistulas was 8.3% during the first year after initial diagnosis of CD, and the cumulative incidence was 15.8% at 10 years after diagnosis. The analysis of the data indicated that the cumulative incidence between years 2 and 10 after the initial diagnosis of CD was 7.5%, which resulted in an annual incidence of 0.83% during this period.^20^ Georgiadou and colleagues conducted a retrospective analysis of a German claims database, which included 13 346 patients with CD, and found that 451 had a concurrent diagnosis of perianal fistula, indicating an overall prevalence of 3.38%.^21^ A meta-analysis of European population-based studies conducted in both Western and Eastern European countries estimated the prevalence of CD-related anal fistulas to be 0.76 per 10 000 population. The estimated annual incidence of CD-related anal fistulas was 0.21 per 10 000 population, based on a median duration of 3.6 years.^22^ Furthermore, a cohort in Taiwan demonstrated that after a 15-year follow-up, the fertilization rate was 14.8%.^23^ Indeed, a systematic review found that the prevalence of anal fistulas among patients with CD in Europe was between 3.4% to 6.0%.^11^ The prevalence of perianal fistulas in CD varies with the disease location, with the lowest incidence occurring in isolated ileal disease (12%) or ileocolonic disease (15%).^11,22,24^ Perianal lesions are most prevalent in colonic disease, occurring in 41% of cases, especially in those with rectal involvement (92%). In 17.2% of patients, perianal lesions can be the first manifestation of CD, occurring more than 6 months before the diagnosis. In 26.9% of cases, the perianal disease presents from 6 months before to 6 months after the diagnosis of CD, while in the remaining 55.9%, the perianal disease is first detected more than 6 months after the diagnosis of CD.^19,25^

## Pathophysiology

 The underlying mechanisms of anal fistulas are not yet fully understood. However, two primary mechanisms appear to play a crucial role: epithelial-to-mesenchymal transition (EMT) and matrix remodelling enzymes. EMT involves the transformation of specialized epithelial cells into mesenchymal-type cells, which can migrate and infiltrate adjacent tissues. This process is essential during embryonic development, organ formation, and wound healing and has also been observed during tumor growth and metastasis. EMT may contribute to the development of fistula-associated neoplasia.^26-28^ Matrix metalloproteinases (MMPs) are enzymes that can break down various components of the extracellular matrix. Increased MMP activity has been observed in both experimental and human IBD.^29-31^ High levels of MMP3 protein and mRNA levels in mononuclear cells and fibroblasts have been detected in the fistula tracts of patients with CD.^32-34^ Along with these theories, the role of microbiota and genetic alterations may also influence the development of anal fistulas.^35,36^

## Classification

 When providing anatomical descriptions of fistulas, it is important to include details such as the type of fistula, the location of both internal and external openings, and any secondary branches and abscesses. The positions of internal and external openings can be described using the “anal clock” system.^37^ The following classification is actually used in clinics.

###  Park’s Classification

 Park’s classification is a surgical-based system that categorizes anal fistulas based on their anatomical location in relation to the sphincter complex.^38^ Despite this classification being introduced some decades ago, it is still commonly used today. The classification includes four types of anal fistulas: intersphincteric, transsphincteric, suprasphincteric, and extrasphincteric.

- intersphincteric (45%) – The fistula penetrates through the internal sphincter but spares the external sphincter.
- Transsphincteric (30%) – The fistula passes through both the internal and external sphincters.
- Suprasphincteric (20%) – The fistula penetrates through the internal sphincter and then extends superiorly in the plane between the sphincters to pass above the external sphincter before extending to the perineum. This classification includes horseshoe abscesses.
- Extrasphincteric (5%) – This fistula is very rare. It forms a connection from the rectum to the perineum that extends laterally to the internal and external sphincter.

The weak point of this classification is that it cannot provide any information regarding the complexity of the fistula (secondary tracts or presence of abscesses) or the presence of proctitis.

###  The St. James’s Classification

 The St. James’s classification is a radiologically-based system that provides a detailed analysis of the primary fistula tract, its relationship to the sphincter, and any secondary tracts and associated abscesses.^39,40^ It categorizes fistulas into five grades: Grade 1, which involves a simple linear intersphincteric tract; Grade 2, which includes an intersphincteric tract with an abscess or secondary tract; Grade 3, which is a transsphincteric tract; Grade 4, which is a transsphincteric tract with an abscess or secondary tract within the ischiorectal fossa; and Grade 5, which involves supralevator and translevator extension.

###  American Gastroenterological Association

 The American Gastroenterological Association proposed a simpler classification system for perianal fistulas, which divides them into two categories: simple or complex.^40^ This classification is based on the anatomy of the fistula tract, the number of external openings, and the presence of abscesses and/or proctitis. This system has prognostic relevance for fistula healing.

 Complex anal fistula is a challenging condition frequently encountered in colorectal surgery. It refers to a type of transsphincteric fistula and encompasses anal fistulas associated with various factors such as malignancy, IBD, radiation, chronic diarrhea, or pre-existing fecal incontinence. The treatment of complex anal fistula poses a significant risk of recurrence and potential incontinence problems due to the diverse causes and variations of the condition. There is no consensus regarding its treatment.^41,42^

## Diagnosis Approach

 The diagnosis of a fistula can typically be established through a comprehensive medical history and physical examination. However, identifying the exact type of fistula may require imaging or examination under anesthesia. Common symptoms include occasional pain, purulent or blood-tinged drainage, recurring abscesses, and seepage. Patients may also report experiencing cycles of swelling, pressure, pain, and spontaneous or planned drainage of an abscess.^5,40,43^

 An experienced physician can detect and accurately classify perianal fistulas and abscesses with very high (90%) accuracy during examination under anesthesia.^5,44^ This approach should be the initial diagnostic method when abscesses are suspected. Imaging techniques can be used subsequently to confirm the appropriate drainage of cavities and to assess fistula anatomy. Sigmoidoscopy and Colonoscopy can evaluate the lower part or full length of the colon. It is important to look for other disorders, especially if UC or CD is suspected.

 Computerized tomography (CT)^45^ is valuable for detecting abscesses and fluid collections that can be drained. It is a fast and easily accessible option in most medical situations. However, it is not as accurate as pelvic magnetic resonance imaging (MRI) in categorizing anal fistulas.^46^ In cases where there is a suspicion of an acute infection in an anal fistula or an underlying abscess, and a prompt diagnosis is crucial, a CT scan might be the most suitable imaging method to quickly diagnose and treat the patient.^47^ In non-hospital settings, CT-fistulography is a helpful and effective way to identify fistula pathways before surgery. Nevertheless, this technique requires experienced radiologists to interpret the images.^48^

 Pelvic MRI is considered the most reliable method for detecting fistula anatomy, complexity, and activity.^49^ Endoanal ultrasonography or 3-dimensional endosonography (EUS) has been proposed as an alternative to MRI, but it requires expertise and has limited accuracy for detecting lesions that are distant from the anal canal.^50,51^ The optimal timing for imaging reassessment is not well-established, so imaging reassessment is typically reserved for patients with unfavourable clinical outcomes. When taking the medical history, it is important to evaluate the patient’s bowel function, continence, obstetric experiences, history of trauma, and previous abscesses.^5,44^

 The differential diagnoses may include other conditions, such as hidradenitis suppurativa, pilonidal disease, skin infections, epidermal inclusion cysts, and Bartholin gland cysts in women.^5,40^ During the physical examination, a rectal examination and anoscopy should be performed to assess the sphincter anatomy and tone before surgery. This examination may also help identify the openings of the fistula.^5,40^

## Endosonography

 EUS is a popular imaging method for assessing the lower rectum, anal sphincters, and pelvic floor. It can be used as an alternative to MRI and provides excellent visualization of the layers of the rectal wall and the anal anatomy. The newer technique of 3-dimensional EUS has shown promising results and can be used to evaluate perianal disease and drain pelvic abscesses. The EUS anatomy of the anal canal is typically described at three levels: upper, middle, and lower anal canal. EUS is particularly useful for identifying internal openings, which are typically located in the subepithelial layer. Fistula tracks may appear differently on examination based on the internal composition or stage of inflammation.^52-54^ A meta-analysis focused on the evaluation of anal fistulas using MRI and EUS revealed that the sensitivities of MRI and EUS were both 87%, while their specificities were 69% for MRI and 43% for EUS.^55^ In this context, transperineal ultrasound (TPUS), as a non-invasive alternative to EUS, has been studied in patients with anorectal abscess, anoperineal fistulas, and rectovaginal fistulas of cryptogenic or CD origin with a sensitivity of 85% and a positive predictive value of 86% for anal fistulas and was of similar value as EUS.^56-58^

## Fistula Activity Assessment

 The purpose of measuring fistula activity is to assess disease severity and response to treatment. To achieve this goal in clinical practice, healthcare providers need to collect data from physical examination, endoscopy, and imaging techniques such as MRI, which provide anatomical details and information on inflammation parameters. There are various clinical and radiological protocols available for this purpose.^45-60^

 Clinical assessment methods include the Perianal Disease Activity Index (PDAI), which evaluates the quality of life and severity of perianal disease based on factors such as fistula discharge, type of perianal disease, and degree of induration, rated on a five-point scale.^61,62^ However, the lack of an established optimal cutoff point for determining a clinically significant response is a major limitation of this index.^63^

 The Anal Disease Activity Index is another modality that uses a linear analogue scale to analyze different symptoms related to anal disease, and it has identified spontaneous pain, pain-limiting locomotion, and pain during defecation as the most reliable parameters for detecting clinical improvement. The fistula drainage assessment is a simple measure of fistula activity and response to medical treatment, which classifies fistulas as either open (draining) or closed (no drainage despite gentle finger compression).^63^

###  MRI

 Pelvic MRI is considered the most reliable imaging method for evaluating fistula characteristics and detecting abscesses.^64,65^ T2-weighted sequences are crucial for identifying the fluid content in fistula tracts or abscesses, while gadolinium-enhanced images may help distinguish pus from granulation tissue in the fistula tract and inflammatory masses. This technique has also been suggested as an important method for evaluating changes after therapy.^37,64^ The van Assche score is the primary MRI index used to assess anal fistulas. This score combines the anatomical characteristics of the fistula with MRI findings related to inflammation.^66^

## Management

 Perianal fistulas frequently lead to substantial deterioration in a patient’s quality of life, marked by perianal pain, swelling, spontaneous discharge of pus, stool, or blood from the fistula opening, and the possibility of fever in cases involving abscess formation. The emergence of severe complications becomes a concern if abscesses give rise to bacterial sepsis. Hence, ensuring proper treatment is a pivotal aspect of managing this condition. The therapeutic approach for perianal fistulas should embrace an interdisciplinary strategy, encompassing both surgical and medical interventions. For complex fistulas involving abscess development, meticulous interdisciplinary coordination is necessary.^66^ The primary treatment objective is to eliminate the infected lesion, establish effective drainage, and encourage fistula closure, all while minimizing harm to the anal sphincter.^67,68^ The integrity of the internal anal sphincter ^47^ and external anal sphincter (EAS) holds paramount importance in preserving normal anal function for patients. Surgical intervention is often a critical consideration in such situations. In essence, numerous experts contend that anal fistulas are unlikely to heal without intervention, and neglecting treatment could lead to the progression of the disease.^69^

## Non-medical Treatment

 The treatment of complex anal fistula is challenging due to its various causes and forms, and it often carries a high risk of recurrence and potential incontinence disorders.^1^ Additionally, there is no agreement among medical professionals on the most effective surgical approach.

 Over the past few decades, various sphincter-sparing techniques have emerged, includingEndorectal advancement flap (ERAF), ligation of inter sphincteric fistula tract (LIFT), fibrin glue, anal fistula plug, fistula laser closure, video-assisted anal fistula treatment (VAAFT), and adipose-derived stem cells.^67-70^

 Several innovative and modified therapies have been proposed and tested in clinical studies in recent years, aiming to reduce the recurrence rate, protect the anal sphincter, and improve postoperative outcomes in patients with anal fistula. These therapies combine independent sphincter-sparing techniques.^70,71^

 However, due to the variety of treatment methods and the inevitable differences in clinical trials, the outcomes of these therapies are variable, which can lead to confusion and misunderstandings.

 The placement of a seton is an established treatment option for individuals with perianal fistulas, as it aids in the drainage or closure of the fistula.^72^ The seton method is a technique that aims to preserve the sphincter muscles by gradually detaching them, leading to fibrosis and necrosis.^1,5^ Over the years, different types of setons, including chemical, cutting, and comfort setons, have been developed. However, the success rate of this method is not satisfactory in patients with complex fistulas or extensive tissue loss or cases where resistance to medication is observed.^73^ This limitation may be ameliorated by combining the seton placement technique with regenerative medicine approaches like stem cell therapy, tissue engineering, and medical treatment.^72-75^ This integration offers a practical and innovative approach to treating perianal fistulas. However, research has indicated that the cutting seton method does not adequately protect the anal sphincter, and the rate of postoperative anal incontinence can be as high as 63%.^67,76^

 The LIFT is a newly introduced technique for treating anal fistula. The technique is implemented by secure closure of the internal and external openings of the tract and removing the infected cryptoglandular tissue.^77^ randomized control trial studies that compared LIFT and conventional open fistulotomy showed that the LIFT procedure is an effective technique with a success rate of 80%, and also LIFT had a lower rate of incontinency and shorter healing time.^77,78^ The LIFT procedure had shown good potential in treating the complex perianal fistula with a satisfactory success rate of more than 75% and a low rate of complication, especially incontinence. Also, It could be repeated with good results.^77-79^ In cases associated with CD, LIFT procedure had good outcomes.^80,81^

 Another modern technique for treating fistulas is Fistula Laser Closure (FiLaC^TM^), which offers the advantage of sphincter preservation. The procedure involves closing the internal opening and then inserting a laser fiber through a catheter into the tract, delivering energy. This method has demonstrated satisfactory short-term and long-term success rates, ranging from 64.1% to 81.1%.^82,83^ A meta-analysis estimated the success rate of FiLaC^TM^ to be around 63%.^84^

 The over-the-scope clip system (OTSC) is a specialized Nitinol clip designed for achieving hemostasis during flexible endoscopy in the gastrointestinal tract.^85,86^ The OTSC has recently been utilized in treating perianal fistulas by applying it to the internal opening.^86,87^ Studies have reported a success rate ranging from 60% to 93.3% when using the OTSC to treat both complex and simple perianal fistulas.^88^

 VAAFT is a new and sphincter-saving technique used to treat complex anal fistulas. It revealed the effectiveness of combining VAAFT with an anal fistula plug, which yielded satisfactory results. The average time for wound healing was 46 days, and none of the patients experienced impaired anal sphincter function.^89,90^ Another study comparing VAAFT with fistulotomy plus seton placement demonstrated positive outcomes for both procedures. However, the VAAFT group exhibited a significantly shorter healing time and a lower postoperative Wexner incontinence score compared with the other group.^91^ Previous studies have reported success rates ranging from 71.2% to 87.1% for VAAFT in complex anal fistulas and 82-84% in anal fistulas associated with CD.^92-94^

 The anal fistula plug is a technique aimed at preserving the sphincter by attempting to close the fistula’s opening primarily.^95,96^ The treatment is known for its simplicity, minimally invasive nature, and relatively short duration.^97^ The procedure involves inserting either a biological or synthetic plug into the fistula tract’s internal opening securing it with a suture attached to its tail.^98^ Long-term follow-ups have shown varying healing rates for this method, ranging from 54% to 80%. The healing rate is inversely proportional to the duration of the follow-up, which typically ranges from 8 weeks to 6.5 months.^99,100^ Additionally, a study involving 84 patients with CD and perianal fistulas reported favourable outcomes with the fistula plug. The method was considered safe with a low risk of incontinence and morbidity. The mean healing rate was approximately 60%, while the recurrence rate was reported to be 13.6%.^101^

 Despite the efficacy of surgery methods in complex fistula, there are no satisfactory effects. Hence, recently, the administration of mesenchymal stem cells extracted from adipose tissue, mainly in association with other treatments, such as the use of fibrin glue, has been introduced. Their primary use in fistulas associated with CD has revealed interesting results. These cells have multi-differentiation abilities such as self-renewal and secrete cytokines to induce regeneration of blood vessels as well as the epithelial layer. This method actually enters phase 3 of the clinical trial, and the primary results show encouraging outcomes.^102^

 The rectal advancement flap (RAF) is a technique aimed at preserving the sphincter and has demonstrated a healing rate ranging from 66% to 87%. It has been suggested as an optimal technique for healing complex cryptoglandular perianal fistulas.^100^ The transanal approach is commonly used to elevate the rectal flap and advance it distally without disrupting the sphincter.^1,40,100^ In a review by Soltani and Kaiser, which included 1654 patients with either CD or cryptoglandular anorectal fistula who underwent RAF, the healing rates were reported as 80.8% with 13.2% incontinence for CD and 64% with 9.4% incontinence for cryptoglandular anorectal fistulas.^103^ Furthermore, it has been proposed that the average recurrence rate for patients with CD who have anorectal fistulas undergoing RAF is approximately 30%.^104,105^

 Recently a new method called photodynamic therapy (PDT) was introduced as a treatment option for anal fistulas.^106,107^ During PDT, light energy is applied, and photosensitizers are used to induce photo-oxidative damage to target tissues or cells. There are two long-term prospective observational trials demonstrating the efficacy of PDT as a sphincter-sparing therapy for anal fistulas. The procedure was found to be simple and safe and resulted in healing rates ranging from 65.3% to 80%. These findings suggest that PDT can be considered as an alternative treatment choice for patients with complex anal fistulas.^106,108^

## Medical Therapies

 The medical treatment of perianal fistula is related to its etiology and elimination of background disease. In any case, all therapeutic approaches should focus on the prevention of septic complications arising from perianal abscess formation. In fact, medical treatments are almost always applied to IBD-related perianal diseases. In this regard, several therapeutic modalities have been evaluated for the treatment of perianal fistulas. However, many of them are not effective well.^7,109^

 A recent systematic review concluded that a combination of medical and surgical treatment approaches is superior to either single treatment alone. The importance of multidisciplinary patient care is highlighted by superior rates of complete remission (52%) in the combination versus single-therapy (43%) group.^110,111^

 Aminosalicylates are not effective in managing PFCD, and corticosteroids are not recommended either.^112-114^ Therefore, these agents cannot be recommended for this indication. It was illustrated that corticosteroids work when combined with cyclosporine, and high recurrence was observed when cyclosporine was discontinued and low-dose corticosteroids continued.

 The utilization of antimicrobial medications such as ciprofloxacin and metronidazole plays a significant role in treating perianal fistulas. This enhances the fistula’s condition and effectively addresses the infectious complications associated with it, as indicated by various sources.^109,112,115^ Nevertheless, there remains a lack of consensus within the guidelines regarding whether these antimicrobials should be employed as primary or secondary treatment options.^115,116^ Existing evidence suggests that when utilized in isolation, the effectiveness of antimicrobials is constrained and thus not advisable.^116^ Consequently, the prominence of this class of medications comes to the forefront when they are used in conjunction with biologics and thiopurines.^109,115^ The combination of ciprofloxacin and adalimumab has demonstrated greater effectiveness compared with adalimumab alone, both in initiating and sustaining remission.^6,7,106,109,117^ Another approach involving a combination of antibiotics is their joint usage with thiopurines. Notably, antibiotics are initially employed in this particular combination. It is worth mentioning that in the context of CD, individuals with mutated NOD2/CARD15 genes are less inclined to respond favorably to antibiotic treatments.^115,117^

 The available evidence for the effectiveness of thiopurines is limited and suggests that they may either have a moderate impact or no significant effect on perianal fistulas in CD when used in isolation. The consensus among most guidelines is that cyclosporine and methotrexate exhibit poor efficacy in this context. Notably, intravenous cyclosporine manages to elicit a response in 88% of patients, with 44% achieving fistula closure.^118^ Among these options, oral tacrolimus stands out as the sole medication within this category that has demonstrated potential effectiveness in inducing a therapeutic response.^117^

 Among all the available medical treatments, the class of anti-tumor necrosis factor (anti-TNF) drugs stands out with the most compelling statistics supporting their effectiveness in addressing perianal fistulas in CD.^45,119^ A meta-analysis assessing the efficacy of various medical interventions for perianal fistulas in CD has revealed that anti-TNF drugs can enhance the rate of achieving therapeutic effects by 1.5 times while also doubling the rates of achieving remission and maintaining a therapeutic response.^117,120^ Nonetheless, one drawback in the role of this drug class in treating perianal fistulas in CD is the elevated risk of relapse after discontinuation.

 Approximately 50% of patients who discontinue the medication are susceptible to experiencing relapse within a span of 5 years.^119,120^ Given this scenario, prolonged administration becomes imperative to sustain remission.^8^ Ustekinumab, classified as a monoclonal antibody, has displayed noteworthy effectiveness in various studies encompassing phases one to three.^3,117^ On the other hand, vedolizumab, another monoclonal antibody, specifically targets the a4b7 integrin, thereby impeding the migration of T-cells into the gastrointestinal tissue.^3,117^ Similar to ustekinumab, vedolizumab holds promise, although its outcomes are still considered experimental.^45,121^ Several combined treatment regimens involving the aforementioned drugs have been explored in this context, leading to improved outcomes.^45^

## Conclusion

 Perianal fistulas pose a common yet intricate challenge due to their engagement with the sphincter complex. Managing complex fistulas necessitates a constant consideration of preserving sphincter functionality. While a range of treatments are available, the absence of a universally effective remedy is evident. Approaches aimed at preserving the sphincter have been evolving, and it remains crucial to regularly assess new techniques before embarking on procedures that could potentially affect continence. Clinicians must remain updated about these advancements to ensure patients have access to sphincter-preserving alternatives.
